# LINC00470 Stimulates Methylation of PTEN to Facilitate the Progression of Endometrial Cancer by Recruiting DNMT3a Through MYC

**DOI:** 10.3389/fonc.2021.646217

**Published:** 2021-06-25

**Authors:** Tiezhong Yi, Yicun Song, Lingling Zuo, Siyun Wang, Jintian Miao

**Affiliations:** ^1^ Department of Gynecology, The First Affiliated Hospital of Harbin Medical University, Harbin, China; ^2^ Department of Pathology, The Fourth Affiliated Hospital of Harbin Medical University, Harbin, China; ^3^ Department of Obstetrics and Gynecology, Heilongjiang Provincial Hospital, Harbin, China

**Keywords:** long intergenic non-protein coding RNA 470, phosphatase and tensin homolog, DNA methylation, endometrial cancer, DNA (cytosine-5)-methyltransferase 3A

## Abstract

**Objectives:**

Increasing researches emphasize the importance of long non-coding RNAs (lncRNAs) in the development of endometrial cancer (EC). There is wide recognition that LINC00470 is a critical participant in the tumorigenesis of cancers such as gastric cancer and glioblastoma, but its possible effects on EC progression remain to be explored.

**Methods:**

We collected EC tissues and cells, where the expression of LINC00470 was determined, and followed by the Kaplan-Meier analysis of EC patient survival. We next examined the effect of LINC00470 and phosphatase and tensin homolog (PTEN) on EC cell migration, invasion, tube formation *in vitro*, and angiogenesis in mice xenografted with tumor after gain- or loss-of-function treatments. RNA pull-down, Co-IP, and ChIP experiments were performed to analyze the targeting relationships among LINC00470, MYC and DNMT3a.

**Results:**

LINC00470 was aberrantly upregulated in EC and its high expression correlated to prognosis of EC patients. LINC00470 promoted invasiveness, migration, and angiogenesis of EC cells, and facilitated tumorigenesis and metastasis *in vivo*, but those effects were reversed by up-regulating PTEN. Functionally, LINC00470 bound to MYC in EC and that LINC00470 stimulated the binding of MYC to DNMT3a, and thus recruited DNMT3a through MYC to promote PTEN methylation.

**Conclusions:**

Our findings revealed that LINC00470 stimulated PTEN methylation to inhibit its expression by MYC-induced recruitment of DNMT3a, thus aggravating EC.

## Background

Endometrial cancer (EC) is the most frequently diagnosed gynecological cancer in developed nations (1). The annual incidence of this gynecological malignancy is increasing, claiming more and more lives (2). Three quarters of patients with EC with early diagnosis have a five-year survival of about 75%, and mainly characterized by the aberrant uterine bleeding and vaginal secretions (3). Elevated risk for EC is associated with age of 50 years, diabetes mellitus, or thyroid diseases, and principally due to excessive estrogen exposure (4). In many cancers, tumor angiogenesis supports the tumor progression due to its close association with tumor metastasis (5). A comprehensive understanding of the pathophysiology of this malignancy is beneficial to facilitate early diagnosis and improve current treatment outcomes (6). Identification of molecules involved in the pathophysiology such as tumor angiogenesis and metastasis is therefore particularly urgent.

Recently, long non-coding RNAs (lncRNAs) have attracted much attention as new therapeutic targets against cancer progression due to their effects on the pathogenesis of various cancers (including gynecological tumors) (7). Specifically, Yan et al. has demonstrated that LINC00470 is associated with PTEN mRNA and suppresses its stability through interaction with the N^6^-methyladenosine (m^6^A) writer METTL3, thereby promoting gastric cancer cell proliferation, migration and invasion (8). LINC00470 could enhance the expression of ELFN2 and play a dominant role in the regulation of GBM cell autophagy to promote the development of glioblastoma (9). These findings suggested that LINC00470 might act as oncogene in EC by manipulating genes. Therefore, we carried out cell and animal experiments to probe into the effects and mechanisms of LINC00470 on angiogenesis and metastasis of EC.

Our initial bioinformatics analysis (available at lncMAP and RNAInter) suggested that LINC00470 may bind to the transcription factor MYC proto-oncogene, bHLH transcription factor (MYC in EC). MYC is a known oncogene in human cancers, whereas its inhibition suppresses tumorigenesis (10). Furthermore, high expression of MYC may predict dismal overall survival rate of EC patients (11). Additionally, MYC can physically interact with DNA (cytosine-5)-methyltransferase 3a (DNMT3a), recruiting it to the promoter of microRNA-200b (miR-200b) to induce DNA hypermethylation (12). DNMT3a is a type of *de novo* methyltransferase responsible for methylating CpG dinucleotides, and its high expression is a factor predictive of poor prognosis of EC patients (13, 14). Phosphatase and tensin homolog (PTEN), a well-known tumor-suppressor, has been reported to be hypermethylated by DNA methyltransferase 1 (DNMT1) in EC (15), while silencing DNMT3a can restore the expression of PTEN, thereby suppressing hepatocellular carcinogenesis (16). Hence, we speculated that LINC00470 could function as an onco-RNA in EC *via* effects on a putative MYC/DNMT3a/PTEN axis. We tested this hypothesis through gain-of-function and loss-of-function experiments and demonstrated the oncogenic functions of LINC00470 both *in vitro* and *in vivo*.

## Materials and Methods

### Ethics Statement

We performed all research protocols with the approval of the Clinical Research Ethics Committee of the First Affiliated Hospital of Harbin Medical University and in accordance with the *Declaration of Helsinki*. All patients had signed informed consent before the experiments. The Animal Ethics Committee of the First Affiliated Hospital of Harbin Medical University approved the animal experiments, which were conducted with care to minimize the number of animals used in the tests and reduce their suffering.

### Bioinformatics Analysis

EC-related lncRNAs were found through the existing literature. The EC-related dataset GSE39099 was downloaded from GEO (https://www.ncbi.nlm.nih.gov/gds), where a box plot was drawn to determine the expression trend of lncRNAs. The significance of differential *p* value of lncRNAs was calculated using t test. LncMAP (Cancer Type: UCEC) (http://bio-bigdata.hrbmu.edu.cn/LncMAP/index.jsp) and RNAInter (http://www.rna-society.org/raid/home.html) were used to predict transcription factors that could bind to lncRNAs. The GEO database tool GEO2R (https://www.ncbi.nlm.nih.gov/geo/geo2r/) was adopted to differentially analyze the EC-related dataset GSE13003 with a total of 45 samples, including 12 normal samples and 28 EC samples. The remaining 5 samples have nothing to do with this study, which were deleted before analysis. Next, the significantly up-regulated genes with logFC > 1 and *p* < 0.01 were screened out. Downstream transcription factors of the lncRNA and significantly differentially expressed genes were intersected to obtain key downstream transcription factors. The subcellular localization of the lncRNAs was predicted by lncATLAS (http://lncatlas.crg.eu) to verify their downstream regulatory mechanisms. With reference to relevant literature, we determined that lncRNAs could affect EC through downstream pathways mediated by key downstream transcription factors and pathways, and then conducted a correlation analysis through Gene Expression Profiling Interactive Analysis (GEPIA) and co-expression analysis through MEM (https://biit.cs.ut.ee/mem/index.cgi) to validate the identified downstream pathways.

### Clinical Tissue Collection

A total of 103 pairs of EC tissue specimens and the matched adjacent normal tissues were obtained from the First Affiliated Hospital of Harbin Medical University from January 2011 to July 2014. Patients with any disease other than EC were excluded, and a gynecological pathologist confirmed the diagnosis of EC and rated the pathological stages as well as histological types according to the 2000 International Federation of Gynecology and Obstetrics (FIGO) standards. Based on the histological classification of the World Health Organization (WHO), histological types were divided into well differentiated (G1; n = 44), moderately differentiated (G2; n = 37), and poorly differentiated (G3; n = 22) carcinomas. All patients were followed up by telephone or subsequent visit, and their 5-year overall survival rate was recorded.

### RNA *In Situ* Hybridization (ISH)

Paraffin-embedded EC tissues were cut into 4-μm-thick sections. A DNA fragment of MATN1-AS1 was cloned into pSPT19 vector (10999644001, Sigma, Aldrich, Shanghai, China) and was adopted to produce the antisense RNA probe according to the protocols of DIG RNA Labeling Kit (SP6/T7) (11175025910, Sigma). The paraffin-embedded sections were dewaxed, treated with Proteinase K (Roche Diagnostics GmbH, Mannheim, Germany), and hybridized with a probe at a concentration of 300 ng/mL for 16 h at 60°C. After that, the sections were incubated with anti-digoxygenin-AP (AP-conjugated Fab fragments) (1:2,000, Roche, #11 093 274 910) for 1 h at 25°C. Coloring reactions were performed with BM Purple in the dark (Roche # 11 442 074 001).

### Cell Line Culture

EC cell lines HEC-1-B (ATCC^®^ HTB-113), HEC-1-A (ATCC^®^ HTB-112), and KLE (ATCC^®^ CRL-1622) were purchased from American Type Culture Collection (ATCC Manassas, VA, USA). HEC-1-B cells were cultured in Dulbecco’s Modified Eagle Medium (DMEM) supplemented with non-essential amino acids (NEAAs), 10% fetal bovine serum (FBS), and 1% penicillin-streptomycin. HEC-1-A cells were grown in McCoy’s 5a (Modified) Medium with 10% FBS. KLE cells were cultured in DMEM/F12 (Nutrient mixture F12) supplemented with 10% FBS and 1% penicillin-streptomycin. Additionally, the immortalized endometrial stromal cell line hEM15A cells (Institute of basic medical sciences CAMS, Beijing, China) were cultured in the mixture of DMEM/F12 Ham’s F12/DME Medium (DME H-16/F-12 50% Mixture w/o HEPES, with NEAA Medium) (1: 1) encompassing 15% FBS. All cells were cultured in an incubator at 37°C with 5% CO_2_. 5-aza-2′-deoxycytidine (5-Aza-CdR; Sigma-Aldrich Chemical Company, St Louis, MO, USA) was diluted in dimethylsulfoxide and used at a concentration of 2 μM.

### Lentivirus Production

To construct lentiviral particles carrying shRNA (sh)-LINC00470, sh-MYC, or sh-PTEN, LINC00470 shRNA was cloned into the pLKO.1 lentiviral plasmid (Addgene) with the sequences shown in **Table S1**. Moreover, to construct a lentivirus overexpressing LINC00470, full-length LINC00470 was cloned into pLV-Puro (VL3001, Invogen Tech. Co., Ltd., Chongqing, China). Lentiviruses overexpressing LINC00470 and the negative control (NC) were from GenePharma (Shanghai, China). The above lentiviral plasmids and packaging vectors were infected into HEK293T cells using Lipofectamine 3000 reagent (L3000015, Invitrogen, Carlsbad, California, USA), followed by collection of the lentiviral particles after 48 h. The cell line was infected with the indicated lentiviruses in the presence of 10 mg/mL polybrene.

### Reverse Transcription-Quantitative Polymerase Chain Reaction (RT-qPCR)

Total RNA was extracted using RNeasy Mini Kit (Qiagen Company, Hilden, Germany). RNA concentration was measured and was then reversely transcribed into the complementary DNA following instructions in the reverse transcription kit (RR047A, Takara Holdings Inc., Kyoto, Japan). qPCR was conducted using a SYBR^®^ Premix Ex TaqTM II (Perfect Real Time) kit (DRR081, Takara) by a real-time quantitative PCR system (ABI7500, BI, Foster City, CA, USA). Primers were synthesized by Shanghai Sangon Biotechnology Co. Ltd., Shanghai, China (primer sequences are shown in **Table S2**). β-actin served as an internal reference, and fold alterations in expression were analyzed with the 2^-Ct^ method.

### Transwell

Invasion and migration assays of the cells were performed using a 24-well Transwell chamber (MAMIC8S10, Merck Millipore, Billerica, MA, USA) coated with/without Matrigel. The resuspended cells (1 × 10^5^ cells/mL) with the serum-free medium were added to the apical side of the chamber, whereas 500 μL medium containing 10% FBS was added to the basolateral side. After 24 h incubation at 37°C with 5% CO_2_, the chamber was fixed with 4% paraformaldehyde for 30 min and stained with 0.5% crystal violet (C3886, Sigma) for 20 min. The invaded/migrated cells were counted under a phase-contrast microscope (Olympus, Tokyo, Japan).

### Western Blot Analysis

The cells were detached by trypsin and lysed with enhanced radio immunoprecipitation assay (RIPA) lysis buffer (Wuhan Boster Biological Technology Co., Ltd., Wuhan, Hubei, China) containing protease inhibitors to extract total protein, and the protein concentration was then determined using the bicinchoninic acid (BCA) protein assay kit (Wuhan Boster Biological Technology Co., Ltd.). Then, the protein was separated by 10% sodium dodecyl sulfate polyacrylamide gel electrophoresis (SDS-PAGE), electrotransferred onto a polyvinylidene fluoride membrane, and blocked with 5% bovine serum albumin (BSA) at room temperature for 2 h. After that, the membrane was probed with diluted anti-rabbit primary antibodies against Vimentin (ab137321, 1: 2000), E-cadherin (ab231303, 1: 500), N-cadherin (ab18203, 1: 1000), MMP-9 (ab76003, 1:1000), MMP-2 (ab92536, 1:1000), vascular endothelial growth factor (VEGFA; ab46154, 1: 1000), MYC (ab32072, 1: 1000), DNMT3a (ab2850, 1: 500), and PTEN (ab32199, 1: 10000) at 4°C overnight. Then, the membrane was re-probed with horseradish peroxidase (HRP)-labeled secondary antibody of goat anti-rabbit antibody (ab205718; 1: 20,000) at room temperature for 1 h, and added with enhanced chemiluminescence (ECL) working fluid (Millipore) for 1 min to develop images. The gray scale of each band was quantified using Image J analysis software in the Western blot image normalized to β-actin (ab8227, 1: 2000). All antibodies were purchased from Abcam (Cambridge, UK).

### Nucleus/Cytoplasm Separation

Cells were resuspended with Hypotonic buffer A (10 mM HEPES (pH7.5), 0.5 mM DTT, 10 mM KCl, and 1.5 mM MgCl_2_) containing protease inhibitor and RNase inhibitor (N8080119, Thermo Fisher Scientific, Rockford, IL, USA). After 10 min incubation on ice, the cells were centrifuged at 1000 × g at 4°C for 10 min, and then the supernatant was collected and further centrifuged at 15,000 × g for 15 min to obtain the cytoplasm. The pellets were first rinsed twice with hypotonic buffer, resuspended with hypotonic buffer B (10 mM HEPES (pH 7.5), 10 mM KCl, 1.5 mM MgCl_2_, 0.5 mM DTT, 0.5% Nonidet P-40), and centrifuged at 6000 × g at 4°C for 10 min. Then the pellets were resuspended again with RIPA buffer (50 mM Tris HCl (pH 7.5), 1500 mM KCl, 1% Nonidet P-40, 0.5% sodium deoxycholate, 0.1% SDS, and 1 mM EDTA pH 8.0) containing protease inhibitor and RNase inhibitor. After 30 min incubation at 4°C, the suspension was centrifuged at 15,000 × g for 20 min, and the obtained supernatant was the nucleus.

### Tube Formation Assay

After reaching 80% cell confluence, EC cells were cultured with serum-free DMEM for another 24 h, and the supernatant was collected as a conditioned medium and stored at -80°C. Each well of a 96-well plate was pre-coated with 50 μL Matrigel (Becton, Dickinson and Company, NJ, USA) for the tube-formation experiment, which was allowed to polymerize for 30 min at 37°C. The endothelial cells were suspended in different conditional media at a density of 1.5 × 10^5^ cells/mL, and 100 μL cell suspension was added to each Matrigel-coated well. An optical microscope (× 400) was used to capture the formation of capillary-like structures after about 6 h.

### RNA Pull-Down Assay

The Pierce ™ Magnetic RNA-Protein Pull-Down Kit (Thermo Fisher Scientific) was used for RNA pull-down experiments in a nuclease-free environment. In brief, cell lysates were prepared using standard IP lysis buffer (Thermo Fisher Scientific). The streptavidin magnetic beads were washed and then bound to the 3’-biotin-labeled LINC00470 probe. Following RNA binding, the cell lysates were incubated with the above beads in protein-RNA binding buffer at 4°C for the binding of RNA to RNA-Binding Proteins (RBP). Then, RBP complexes were released from the beads through stringent washing and elution, and the retrieved samples were collected for Western blot analysis.

### Co-Immunoprecipitation (Co-IP)

Transfected cells were lysed in lysis buffer (50 mM Tris-HCl (pH 7.4), 150 mM NaCl, 10% glycerol, 1 mM ethylene diamine tetraacetic acid (EDTA), 0.5% NP-40 and protease inhibitor mixture), followed by centrifugation to remove cell debris. The cleared cell lysates were incubated with antibodies to MYC (ab32072, 5 µg/mL), DNMT3a (ab2850, 2 µg/mL) and 15 μL protein A/G beads (Santa Cruz Biotechnology, Inc, Santa Cruz, CA, USA) for 2 h. After extensive washing, the beads were boiled at 100°C for 5 min. The proteins were separated by SDS-PAGE and then transferred onto nitrocellulose membrane (Millipore) for subsequent immunoblotting.

### Chromatin Immunoprecipitation Assay (ChIP)

Cells were fixed with formaldehyde for 10 min to produce DNA-protein cross-links. Then an ultrasonic disruptor was used to disrupt the cells and sonicate chromatin into fragments. The fragments were probed with IgG antibody (ab172730, 1: 1000, Abcam) and DNMT3a antibody (ab2850, 2 µg/mL) at 4°C overnight. The DNA-protein complexes were precipitated with Protein Agarose/Sepharose and then centrifuged at 12,000 g for 5 min, and the supernatant discarded. The non-specific complexes were washed and de-crosslinked at 65°C overnight followed by addition of phenol/chloroform to purify and recover the DNA fragments. Finally, RT-qPCR was performed to detect the binding of DNMT3a with PTEN-specific primers, which are shown in **Table S3**.

### DNA Extraction and Methylation-Specific PCR (MSP)

DNA was extracted with Qiagen DNeasy tissue kit (Qiagen) and 1 µg samples of DNA were mixed into 100 µL water and denatured through the addition of 7 µL 3 M NaOH at 37°C for 10 min. Each denatured DNA solution was then added with 550 µL freshly prepared sodium bisulfite mixture (Qiagen, Hilden, Germany). The obtained mixtures were then incubated at 50°C for 16 h. During bisulfite modification, unmethylated cytosines were deaminated and converted to uracils, whereas 5-methylcytosine remained unchanged. DNA samples were then purified by ethanol precipitation and resuspended in 25-50 µL TE buffer (10 mM Tris/0.1 mM EDTA, pH 7.5). The bisulfite-treated DNA was amplified with methylation specific primers (annealed at 60°C for 40 cycles) or unmethylated-specific primers (U) (annealed at 58°C for 40 cycles). The primer sequences were M-F: GTATTTCGAGTAAAGGAAGAAGACG, M-R: GATAAAAAACTACAACCCAACGAA, U-F: TATTTTGAGTAAAGGAAGAAGATGA, and U-R: CAATAAAAAACTACAACCCAACAAA.

### Detection of Tumor Formation and Metastasis in Nude Mice

Six-week-old female BALB nude (nu/nu) mice purchased from SJA Laboratory Animal Co., Ltd. (Hunan, China; n = 48) were kept in specific pathogen-free environment. Xenograft tumors were produced by subcutaneous injection of the KLE cells (2 × 10^6^) with sh-LINC00470 or sh-PTEN. The tumor volume was measured with calipers every week and calculated as follows: V = 0.5 × L × W^2^, where L and W represents tumor length and width. Four weeks later, the mice were euthanized, their tumors were excised and photographed, and their tissue sections were obtained for further immunohistochemical staining. To develop the tumor metastasis nude mouse model, EC cells with sh-LINC00470 or sh-PTEN (50 µL each) were injected into the lumen of the uterine horn of the mice (17). After 7 weeks, the mice were sacrificed, their lungs excised and photographed, and the number of tumor nodules visible on the lung surface were counted.

### Immunohistochemistry

Paraffin-embedded tissue sections were deparaffinized and hydrated. Endogenous peroxidase activity was blocked by incubation with 0.3% H_2_O_2_ for 30 min at 37°C. After washing with PBS, the tissue sections were boiled in 10 mM citrate buffer (pH 6.0) for 30 min. Once cooled to room temperature, the tissue sections were blocked with 5% normal goat serum at 37°C for 1 h, and then incubated with CD34 (ab81289, 1: 2500) or PTEN (ab170941, 1: 100) antibodies at 4°C overnight. After washing 3 times with PBS, the tissue sections were incubated with secondary antibody IgG (ab205718; 1: 2000) at 37°C for 1 h. After washing three times in PBS, the tissue sections were then incubated with HRP-conjugated streptavidin (1: 1000 diluted) at 37°C for 45 min. Newly prepared DAB was added for color development, and all the tissue sections were counterstained with hematoxylin. Finally, the stained tissue sections were analyzed under an OLYMPUS BX51 microscope.

### Statistical Analysis

All data were processed using the SPSS 21.0 version (IBM Corp., Chicago, IL, USA), and measurement data were expressed as mean ± standard deviation. Independent sample *t-*test was used for data comparison between groups and one-way analysis of variance (ANOVA) and Tukey’s *post hoc* test were used for data comparison between multiple groups. Data between groups at different time points were compared using two-factor ANOVA followed by Bonferroni’s *post hoc* test. Pearson correlation was used to analyze the relationship between LINC00470 and PTEN. Kaplan-Meier was adopted to calculate the patient survival curve, and log-rank analysis was applied to analyze the difference in patient survival. *p* < 0.05 indicates that the difference is statistically significant.

## Results

### LINC00470 Is Highly Expressed in EC and Was Associated With Prognosis

**Figure 1:**
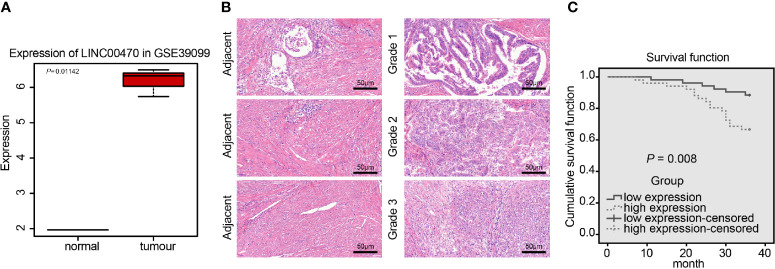
LINC00470 was overexpressed in EC and related to the poor prognosis of EC patients. **(A)** Expression box diagram of LINC00470 in the dataset GSE39099 (left panel); RT-qPCR determination of the LINC00470 expression in 103 EC tissues and adjacent normal tissues (right panel). **(B)** The expression of LINC00470 in EC tissues and adjacent normal tissues assessed by ISH, scale bar = 50 μm (left panel); the pathological changes of EC tissues and adjacent normal tissues assessed by HE staining (right panel), scale bar = 50 μm. **(C)** Kaplan-Meier analysis of the prognosis of EC patients with high or low expression of LINC00470. Data comparison between two groups were performed using independent sample *t-*test.

LINC00470 has been reported to be highly expressed in gastric cancer (8) and glioblastoma (9, 18), but has not been proven to be related to EC. To analyze whether LINC00470 is associated with EC, we adopted the dataset GSE39099 to extract the expression data of LINC00470 and found that LINC00470 was significantly highly expressed in EC (**Figure 1A**). Subsequently, we determined the LINC00470 expression in EC tissues and their matched adjacent normal tissues using RT-qPCR, which showed that LINC00470 was highly expressed in the tissues of Grade 1, 2, and 3 EC compared with that in adjacent normal tissues, among which LINC00470 showed the highest expression in the tissues of Grade 3 EC tissues, the most invasive EC tissues (**Figure 1A**). Further, our ISH results revealed that LINC00470 was poorly expressed in adjacent normal tissues, but strongly expressed in the tissues of different grade ECs. In addition, HE staining was performed on EC and adjacent tissues to detect the degree of lesions. It was found that the adjacent tissues had clear papillary and micropapillary structures, but the EC tissues of different grades exhibited no obvious papillary and micropapillary structures, accompanied by serious cell proliferation (**Figure 1B**). Finally, patients were assigned into high and low LINC00470 expression groups with the median expression of LINC00470 in the RT-qPCR results as a cutoff value. Kaplan-Meier analysis suggested that EC patients with high expression of LINC00470 had relatively poor survival rates (**Figure 1C**). The above results revealed overexpressed LINC00470 in EC and its association with the poor prognosis of EC patients.

### Silencing LINC00470 Inhibits the Invasive/Migratory Capacity and Angiogenesis of EC Cells *In Vitro*

**Figure 2:**
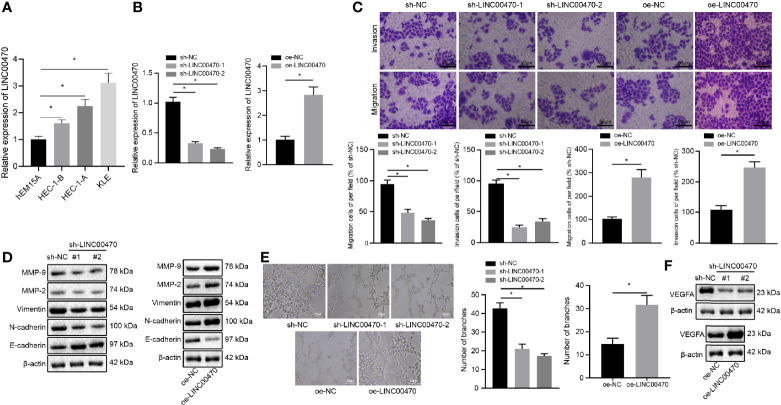
Silencing LINC00470 inhibited the invasion/migration potentials and angiogenesis of EC cells. **(A)** RT-qPCR determination of the expression of LINC00470 in immortalized endometrial stromal cell line epithelial cell line hEM15A, and HEC-1-B, HEC-1-A, and KLE cells. KLE cells with the highest expression of LINC00470 were selected for subsequent loss-of-function experiments and LINC00470 was silenced in KLE cells while HEC-1-B cells with lowest expression of LINC00470 were selected for gain-of-function experiments and LINC00470 was overexpressed in HEC-1-B cells. **(B)** RT-qPCR determination of the expression of LINC00470 in the KLE cells after LINC00470 knockdown and in HEC-1-B cells after LINC00470 overexpression. **(C)** Changes in migration and invasion ability assessed by Transwell in the KLE cells after LINC00470 knockdown and in HEC-1-B cells after LINC00470 overexpression, scale bar = 50 μm. **(D)** Western blot analysis of the expression of Vimentin, E-cadherin, N-cadherin, MMP-9, and MMP-2 in the KLE cells after LINC00470 knockdown and in HEC-1-B cells after LINC00470 overexpression normalized to β-actin. **(E)** The tube formation ability of the cells assessed by tube formation experiment after LINC00470 knockdown or overexpression, scale bar = 50 μm. **(F)** Western blot analysis of VEGFA expression in the KLE cells after LINC00470 knockdown and in HEC-1-B cells after LINC00470 overexpression normalized to β-actin. The experimental data were expressed as mean ± standard deviation, and data comparison between multiple groups were conducted with one-way ANOVA and Tukey’s *post hoc* test, *p < 0.05.

To detect the expression of LINC00470 in the immortalized endometrial stromal cell line hEM15A and EC cell lines HEC-1-B, HEC-1-A, and KLE, we performed RT-qPCR, which showed that LINC00470 was highly expressed in EC cell lines (**Figure 2A**). Then, KLE/HEC-1-B cells with the highest/lowest expression of LINC00470 were selected for subsequent experiments. Further, RT-qPCR results showed that treatment with sh-LINC00470-1 and sh-LINC00470-2 reduced the expression of LINC00470 in KLE cells, and treatment with oe-LINC00470 enhanced the expression of LINC00470 in HEC-1-B cells, which validated the successful infection of LINC00470 (**Figure 2B**). Then, our Transwell results revealed that the migration and invasion abilities of EC cells were significantly weakened after down-regulation of LINC00470 but noticeably strengthened after overexpressing LINC00470 (**Figure 2C**). Additionally, Western blot analysis unraveled that downregulation of LINC00470 decreased the expression of Vimentin, N-cadherin, MMP-9, and MMP-2 but increased the E-cadherin expression, while opposite trends were observed upon overexpression of LINC00470 (**Figure 2D**). Tumor vascularization due to angiogenesis reportedly plays an important role in tumor progression and metastasis, and its degree relates to tumor grade and aggressiveness (19). Thus, we further examined the role of LINC00470 in tumor vascularization. Accordingly, our results of the tube formation experiment *in vitro* verified that down-regulation of LINC00470 significantly reduced tube formation ability, while LINC00470 overexpression markedly promoted the tube formation ability (**Figure 2E**). Moreover, our Western blot results of the expression of VEGFA uncovered that the expression of VEGFA was significantly reduced after LINC00470 knockdown, but was significantly increased after overexpressing LINC00470 (**Figure 2F**).

### LINC00470 Knockdown Inhibits Angiogenesis and Metastasis of EC Cells *In Vivo*

**Figure 3:**
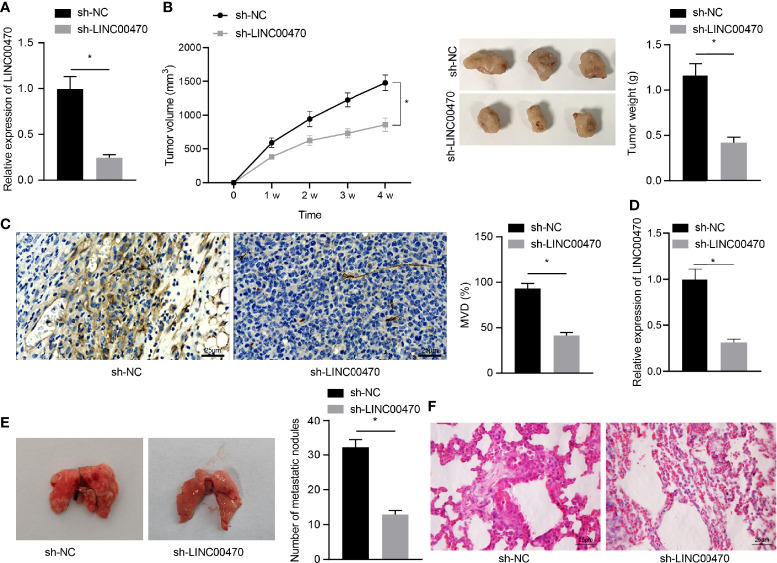
Depletion of LINC00470 could restrain the angiogenesis and metastasis of EC cells *in vivo*. Nude mouse models of tumor formation and metastasis were established through injection of KLE cells infected with lentivirus carrying sh-NC or sh-LINC00470. **(A)** RT-qPCR determination of the expression of LINC00470 in the tumor tissues of mice from the tumor formation model. **(B)** Growth curves, solid maps, and weight of the tumors. **(C)** Microvessel density, scale bar = 25 μm. **(D)** LINC00470 expression in tumor tissues of mice from the tumor metastasis model determined by RT-qPCR. **(E)** Visual observation of lung metastases and counting of metastatic nodules. **(F)** Metastasis of lung tissue assessed by HE staining, scale bar = 25 μm. The experimental data were expressed as mean ± standard deviation, *t*-test was used for data comparison between two groups, and repeated measures ANOVA was used for comparison between different time points followed by Bonferroni’s *post hoc* test, *p < 0.05, N = 12.

To further examine the role of LINC00470 *in vivo*, we first infected KLE cells with lentiviral particles carrying sh-NC or sh-LINC00470 and subcutaneously injected KLE cells with lentivirus into nude mice to construct a xenograft mouse EC model. Initially, RT-qPCR showed that the expression of LINC00470 was significantly reduced by treatment with lentivirus expressing sh-LINC00470 (**Figure 3A**). Besides, the growth rate and weight of tumors as well as microvessel density in the tumor tissues were significantly reduced after LINC00470 knockdown (**Figures 3B, C**). Meanwhile, the tumor metastasis mouse model was constructed with injection of KLE cells infected with sh-LINC00470 or sh-PTEN into the lumen of the uterine horn of the mice. Tumor tissues were collected from each mouse, and the expression of LINC00470 in tumor tissues was determined by RT-qPCR. The results showed that, compared with the sh-NC, the expression of LINC00470 in tumor tissues was decreased in response to sh-LINC00470 (**Figure 3D**). To detect the effect of LINC00470 on cancer metastasis *in vivo*, we injected EC cells with sh-LINC00470 into the lumen of uterine horn of nude mice, and then metastatic tumors in the lung was observed. The obtained results revealed that the number of lung metastatic nodules was significantly decreased after depletion of LINC00470 (**Figures 3E, F**). The above result indicated that silencing of LINC00470 could inhibit the angiogenesis and metastasis of EC cells *in vivo*.

### LINC00470 Binds to MYC Protein in EC and Promotes the Binding of MYC to DNMT3a

**Figure 4:**
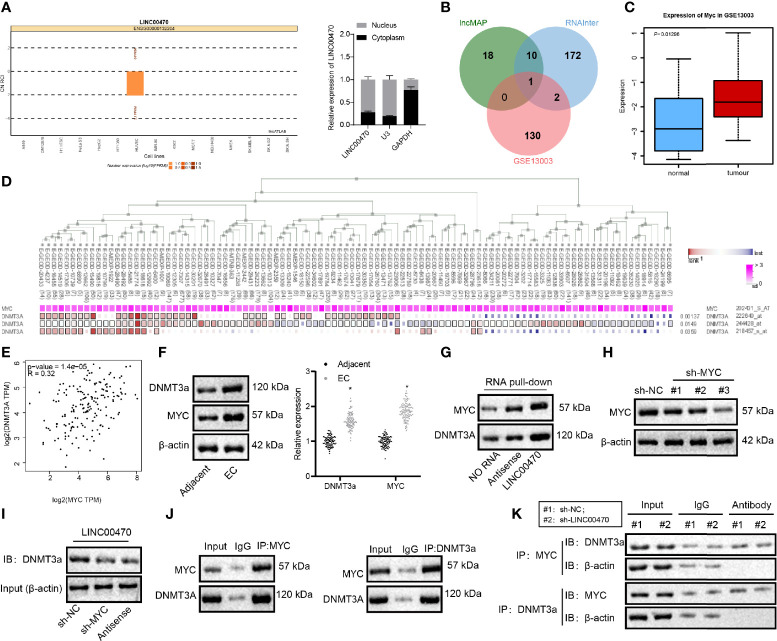
LINC00470 bound to MYC protein in EC and strengthened the binding of MYC to DNMT3a. **(A)** The subcellular localization of LINC00470 predicted by LncATLAS and the expression distribution of LINC00470 in the cytoplasm and nucleus assessed by nucleus/cytoplasm separation and RT-qPCR. **(B)** Venn diagram displaying the intersection between transcription factors bound to LINC00470 predicted by LncMAP and RNAInter and the significantly upregulated genes obtained by GSE13003; the intersected gene was MYC. **(C)** The expression box plot of MYC in the dataset GSE13003. The red box indicates the expression in EC samples, and the blue box indicates the expression in normal samples. **(D)** The significant co-expression between MYC and DNMT3a acquired by MEM analysis. **(E)** The correlation between the expression of MYC and DNMT3a in EC obtained by GEPIA analysis. **(F)** The mRNA expression of MYC and DNMT3a in EC tissues and adjacent normal tissues (n = 103) determined with RT-qPCR (left panel); Representative images of Western blots of MYC and DNMT3a proteins in EC tissues and adjacent normal tissues (n = 103) normalized to β-actin (right panel). **(G)** The binding of LINC00470 to MYC and DNMT3a assessed by RNA pull-down assay. **(H)** Western blot analysis of the expression of MYC in KLE cells after sh-MYC-1, sh-MYC-2, or sh-MYC-3 treatment normalized to β-actin, and sh-MYC-3 with the highest silencing efficiency were selected for subsequent experiments. **(I)** Changes in the binding of sense-LINC00470 to DNMT3a detected by RNA pull-down assay. **(J)** The binding of MYC and DNMT3a assessed by Co-IP. **(K)** The binding of MYC to DNMT3a in KLE cells after sh-LINC00470 treatment examined by Co-IP. Cellular experiment was independently repeated 3 times.

To investigate the underlying mechanism of LINC00470 on EC, we adopted the lncATLAS analysis, which predicted a nuclear localization of LINC00470 (**Figure 4A**). Consistent with the ISH results (**Figure 1B**), our results from nucleus/cytoplasm separation and RT-qPCR confirmed that LINC00470 was mainly located in the nucleus of EC cells (**Figure 4A**), suggesting that LINC00470 may regulate downstream genes through transcription factors. Therefore, we first looked for transcription factors likely binding to LINC00470 and thus playing a critical role in EC. To this end, the lncMAP and RNAInter websites predicted 29 and 185 downstream transcription factors, respectively. Then, we obtained 133 significantly up-regulated genes from the dataset GSE13003, which was intersected with the downstream transcription factors and finally MYC was obtained (**Figures 4B, C**). Previous evidence has demonstrated that high expression of MYC is related to low overall survival rate of EC patients (11). Therefore, we speculated that LINC00470 may regulate downstream pathways through MYC. Additionally, our data obtained through MEM analysis revealed that MYC and DNMT3a had a significant co-expression relationship (**Figure 4D**). Indeed, MYC can bind to the corepressor DNMT3a and correlate with DNA methyltransferase activity (20, 21). Furthermore, existing literature has shown that MYC physically interacts with DNMT3a, recruits DNMT3a to gene promoters to inhibit gene expression, and promotes DNA methylation (12, 22). The GEPIA analysis also suggested a significant positive correlation between MYC and DNMT3a expression in EC (**Figure 4E**). Through bioinformatics analysis, it was predicted that LINC00470 might exert function on EC progression by regulating MYC and DNMT3a.

In the subsequent analysis, RT-qPCR and Western blot analysis validated that MYC and DNMT3a mRNA and protein expression were significantly enriched in EC tissues compared with the adjacent normal tissues (**Figure 4F**). Besides, RNA pull-down assay revealed that LINC00470 could pull down MYC and DNMT3a proteins (**Figure 4G**). After silencing MYC in KLE cells, we measured the amount of DNMT3a protein in the magnetic beads bound with a 3’-biotin-labeled LINC00470 probe. RNA pull-down experiment results revealed that the expression of MYC was significantly reduced after silencing MYC (**Figure 4H**) and that the protein expression of DNMT3a pulled down by LINC00470 probe was significantly reduced after silencing MYC (**Figure 4I**). Co-IP was conducted to detect the expression of MYC in the DNMT3a complex precipitated by DNMT3a antibody in EC cells, which showed that MYC and DNMT3a proteins could bind to each other (**Figure 4J**), although the binding of MYC to DNMT3a was significantly inhibited after silencing LINC00470 (**Figure 4K**). The above results revealed that LINC00470 bound to MYC protein in EC and facilitated the binding of MYC to DNMT3a.

### LINC00470 Recruits DNMT3a Through MYC to Promote PTEN Methylation

**Figure 5:**
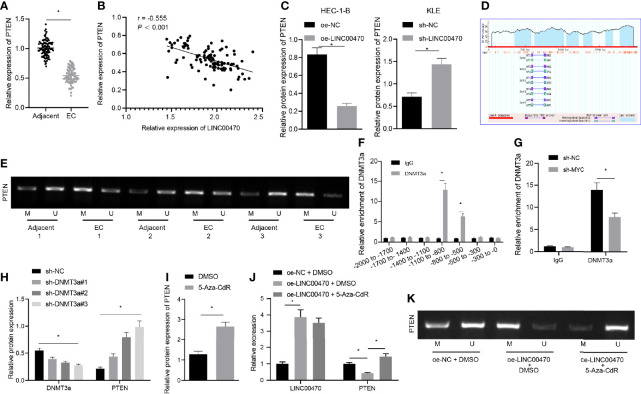
LINC00470 recruited DNMT3a through MYC to stimulate PTEN methylation and down-regulate PTEN expression. **(A)** RT-qPCR determination of PTEN expression in 103 EC tissues and 70 normal control tissues. **(B)** Correlation analysis between LINC00470 and PTEN in EC. **(C)** Western blot analysis of PTEN expression in HEC-1-B and KLE cells after oe-LINC00470 or sh-LINC00470 treatment normalized to β-actin. **(D)** Rich CpG islands in gene promoters predicted by Methprimer (http//www.urogene.org/cgi-bin/methprimer/methprimer.cgi). **(E)** MSP determination of the methylation status of PTEN in EC tissues, wherein U means non-methylation and M means methylation. **(F)** The binding position of DNMT3a in the PTEN promoter region assessed by ChIP. **(G)** Changes of DNMT3a in PTEN gene promoter region after sh-MYC treatment detected by ChIP. **(H)** Western blot analysis of the expression of DNMT3a and PTEN in the cells after sh-DNMT3a-1, sh-DNMT3a-2, or sh-DNMT3a-3 treatment normalized to β-actin. **(I)** Western blot analysis of the expression of PTEN in the cells after 5-Aza-CdR treatment normalized to β-actin. **(J)** RT-qPCR measurement of LINC00470 and PTEN in HEC-1-B cells after oe-LINC00470 or 5-Aza-CdR treatment. **(K)** MSP assessment of the methylation status of PTEN in the methylation status of PTEN after oe-LINC00470 or 5-Aza-CdR treatment. The experimental data were expressed as mean ± standard deviation, and the comparison between two groups was performed using independent sample *t-*test. The data between multiple groups were compared using one-way ANOVA and Tukey’s *post hoc* test, and Pearson correlation coefficient was used to analyze the correlation between two groups. *p < 0.05.

To further determine the downstream mechanism of LINC00470/MYC/DNMT3a axis, we resorted the previous references. DNMT3a protein binds around the promoter region of PTEN gene, while DNMT3a silencing induces demethylation of the PTEN promoter (16, 23). Besides, DNMT3a can inhibit the expression of PTEN through methylation of the PTEN promoter (24). Importantly, PTEN is significantly under-expressed in EC tissues and cells (25). Therefore, we assumed that LINC00470 regulated the expression of PTEN by binding to DNMT3a and MYC. To validate this assumption, we performed RT-qPCR and Western blot analysis, which showed that PTEN was significantly under-expressed in EC tissues in contrast to adjacent normal tissues (**Figure 5A**), and revealed that LINC00470 expression was negatively correlated with PTEN expression in EC tissues (**Figures 5B, C**). To study this pathway in depth, we made predictions from Methprimer (http://www.urogene.org/cgi-bin/methprimer/methprimer.cgi), which revealed that the gene promoters were rich in CpG islands (**Figure 5D**). We then adopted MSP to detect the methylation status of PTEN in EC tissues, whose results showed that PTEN was highly methylated in EC tissues (**Figure 5E**). Further, our ChIP results revealed that DNMT3a may bind to -800 to -500 and -1100 to -800 nucleotide fragments of the PTEN promoter region (**Figure 5F**). After silencing MYC, the binding of DNMT3a to the PTEN promoter decreased significantly (**Figure 5G**). In an effort to further confirm the regulation of PTEN expression by DNMT3a, we silenced DNMT3a and measured the expression of DNMT3a and PTEN using Western blot analysis. The obtained data showed that the expression of PTEN was significantly elevated after silencing DNMT3a (**Figure 5H**). After the cells were treated with 5-Aza-CdR to inhibit DNA methylation, the expression of PTEN was assessed by Western blot analysis, and the results revealed that the expression of PTEN was significant elevated after 5-Aza-CdR treatment (**Figure 5I**). RT-qPCR was used to measure the expression of LINC00470 and PTEN (**Figure 5J**), whereas MSP was performed to detect the methylation status of PTEN (**Figure 5K**) in HEC-1-B cells treated with oe-LINC00470 or 5-Aza-CdR. The results demonstrated that overexpression of LINC00470 significantly reduced the expression of PTEN and PTEN was hypermethylated, which was reversed by 5-Aza-CdR treatment. The above results supported our speculation that LINC00470 recruited DNMT3a *via* MYC to promote PTEN methylation and inhibit PTEN expression.

### Knockdown of LINC00470 Inhibits EC Cell Invasion/Migration and Angiogenesis Through PTEN

**Figure 6:**
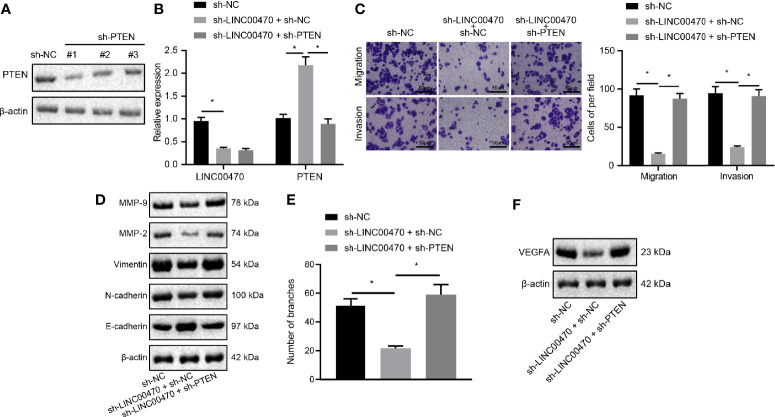
Silencing of LINC00470 inhibited the invasive and migratory capacities of EC cells and angiogenesis through up-regulating PTEN. **(A)** Western blot analysis of the expression of PTEN in KLE cells after sh-PTEN-1, sh-PTEN-2, or sh-PTEN-3 treatment normalized to β-actin. **(B)** RT-qPCR determination of LINC00470 and PTEN expression after sh-LINC00470 or sh-PTEN treatment. **(C)** Changes in cell migration and invasion ability after sh-LINC00470 or sh-PTEN treatment assessed by Transwell (scale bar = 50 μm). **(D)** Western blot analysis of the Vimentin, E-cadherin, N-cadherin, MMP-9, and MMP-2 expression after sh-LINC00470 or sh-PTEN treatment normalized to β-actin. **(E)** Cell tube formation ability assessed by tube formation experiment. **(F)** Western blot analysis of the expression of VEGFA after sh-LINC00470 or sh-PTEN treatment. The experimental data were expressed as mean ± standard deviation, and the comparison between two groups were conducted using independent sample *t*-test. One-way ANOVA and Tukey’s *post hoc* test were used for data comparison among multiple groups, *p < 0.05.

To further examine the regulation of LINC00470 through PTEN in EC, we infected sh-NC, sh-PTEN-1, sh-PTEN-2, or sh-PTEN-3 into KLE cells. First, the expression of PTEN was measured by Western blot analysis, and the sh-PTEN-1 with the highest silencing efficiency was selected for subsequent experiments (**Figure 6A**). Then, our RT-qPCR results showed that down-regulation of LINC00470 increased the expression of PTEN, which was reversed by further silencing PTEN (**Figure 6B**). Further, our experimental results of Transwell and Western blot analysis revealed that silencing LINC00470 alone significantly inhibited the invasion and migration potentials of cells, and reduced the expression of Vimentin, N-cadherin, MMP-9, and MMP-2, but increased the expression of E-cadherin, all of which could be reversed by further silencing of PTEN (**Figures 6C, D**). Next, we showed that silencing LINC00470 alone significantly inhibited the tube formation ability of the cells and reduced the expression of VEGFA, which was rescued by simultaneously silencing PTEN (**Figures 6E, F**). The aforementioned evidence supported that LINC00470 knockdown inhibited the invasion/migration and angiogenesis of EC cells *via* inhibition of PTEN.

### LINC00470 Promotes Angiogenesis and Metastasis of EC Cells *In Vivo* by Inhibiting PTEN

**Figure 7:**
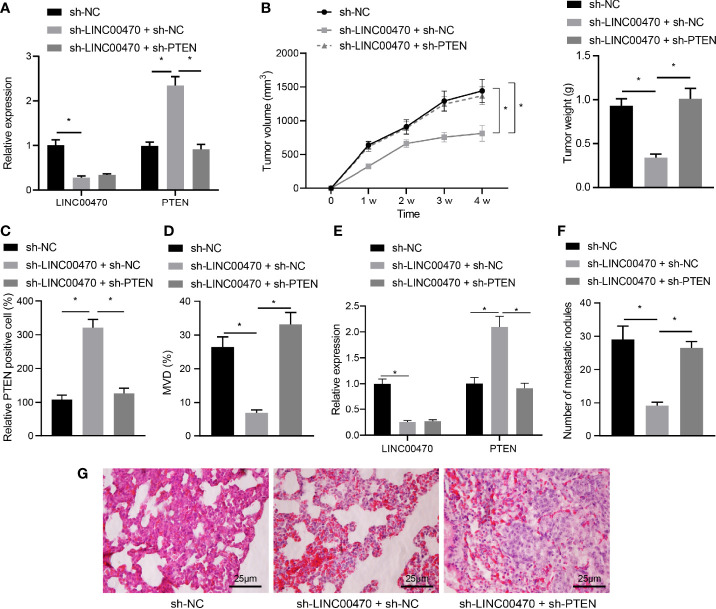
Silencing of LINC00470 inhibited angiogenesis and metastasis of EC cells *in vivo* through PTEN. After knocking down LINC00470 and PTEN with lentivirus in KLE cells, the cells were injected to construct nude mouse tumor formation and metastasis models. **(A)** RT-qPCR determination of the expression of LINC00470 and PTEN in tumor tissues of mice from the tumor formation model. **(B)** Growth curve, solid map, and weight of tumors after knocking down LINC00470 and PTEN. **(C)** The expression of PTEN in tumor tissues after knocking down LINC00470 and PTEN assessed by IHC. **(D)** Microvessel density after knocking down LINC00470 and PTEN. **(E)** LINC00470 expression in tumor tissues of mice from the tumor metastasis model determined by RT-qPCR. **(F)** Visual observation of lung metastasis and counting of nodules after knocking down LINC00470 and PTEN. **(G)** Lung metastasis after knocking down LINC00470 and PTEN examined by HE staining, scale bar = 25 μm. The experimental data were expressed as mean ± standard deviation, and *t*-test was used for data comparison between two groups. One-way ANOVA and Tukey’s *post hoc* test were used for comparison among multiple groups, and repeated measures ANOVA was used for data comparison at different time points followed by Bonferroni’s *post hoc* test. *p < 0.05, N = 12.

To further explore the regulatory effect of LINC00470 on EC through PTEN *in vivo*, we knocked down LINC00470 and PTEN with lentivirus in KLE cells, which were then injected subcutaneously injected in nude mice to construct an EC model. First, the results of our RT-qPCR and *in vivo* EC cell formation tests showed that down-regulation of LINC00470 in tumor tissues significantly increased the expression of PTEN, while this increase was reversed in the tumor tissues of nude mice injected with LINC00470-deficient and PTEN-deficient KLE cells (**Figure 7A**). LINC00470 knockdown reduced tumor volume and growth rate, which could be reversed by knockdown of PTEN (**Figure 7B**). Then, our IHC results exhibited that depletion of LINC00470 significantly increased the expression of PTEN in tumor tissues but reduced the microvessel density, which could be recovered by down-regulating PTEN (**Figures 7C, D**).

To detect the effect of LINC00470 on cancer metastasis *in vivo*, EC cells infected with lentivirus expressing sh-NC, sh-LINC00470 + sh-NC or sh-LINC00470 + sh-PTEN (50 µL each) were injected into the lumen of the uterine horn of the mice. Tumor tissues were collected from the mice and the expression of LINC00470 was determined by RT-qPCR. The results showed that compared with sh-NC group, LINC00470 expression was reduced, while PTEN mRNA expression was increased in tumor tissues of mice injected with sh-LINC00470 + sh-NC-infected cells. Further treatment of silencing of PTEN decreased PTEN mRNA expression without significant change in LINC00470 expression compared with treatment with sh-LINC00470 + sh-NC (**Figure 7E**). Through observation of lung metastasis using HE staining, we discovered that lung metastatic nodules were significantly reduced in number after knockdown of LINC00470, which could be reversed by the further silencing PTEN (**Figures 7F, G**). The above results revealed that LINC00470 facilitated angiogenesis and metastasis of EC cells *in vivo* by inhibiting PTEN.

## Discussion

EC is a common gynecologic tumor with increasing prevalence in the clinic and difficulties in its management (1, 6). The diverse regulatory mechanisms of lncRNAs regulating gene expression include transcriptional/post-transcriptional regulation and epigenetic modifications during the process of RNA transcription (25). Several lncRNAs exhibit specifically altered expression profiles in EC tissues compared to normal adjacent tissues (7), serving as critical players in EC initiation and development. Here, we investigated the role of LINC00470 in EC, and we provided evidence that LINC00470 recruited DNMT3a through MYC to stimulate PTEN methylation and decrease PTEN expression, thereby contributing to the aggressive and metastatic phenotypes of EC cells (**Figure S1**).

In our present study, we first found highly expressed LINC00470 in EC cells and tissues and then experimentally confirmed that aberrant upregulation of LINC00470 was associated with poor prognosis of patients with EC. In agreement with our findings, the LINC00470 expression was noticeably enhanced in gastric cancer tissues and cell lines, which was likewise associated with poor prognosis, distant metastasis, and tumor-node-metastasis stage (8). Additionally, LINC00470 plays a vital role in the tumorigenesis of glioblastoma and its high expression correlates with poor prognosis of patients with glioblastoma (18). Furthermore, LINC00470 can promote malignant behaviors of hepatocellular carcinoma cells (26), supporting the oncogenic effect of LINC00470 in cancers. LncRNAs play an important role in the occurrence and development, clinical medication and prognosis of EC. For example, lncRNA colon cancer-associated transcript 2 (CCAT2) is also reported to serve as an oncogene and its depletion suppresses cell growth and metastasis of EC cells *via* PTEN/PI3K/AKT and mTOR axis by binding to miR-216b (27). The similar mechanism was reported on lncRNA DLEU1, which combined with mTOR to activate the PI3K/AKT/mTOR pathway to promote EC progression (28). Conversely, elevation of MIR22HG could suppress EC cell proliferation and arrested EC cells in G0/G1 phase by negatively regulating miR-141-3p (29). Moreover, LINC00672 overexpression inhibits the development of malignant phenotypes of EC both *in vitro* and *in vivo* by inhibiting LASP1 expression (30).

The next findings in this study revealed that LINC00470 not only bound to MYC protein in EC but also enhanced the binding of MYC to DNMT3a. Besides, MYC recruited DNMT3a to stimulate PTEN methylation. As reported by Lee et al., the oncogenic role of MYC has been demonstrated in EC, where its overexpression was associated with a short overall survival of patients with EC (11). Likewise, it has been reported that higher DNMT3a expression correlates with a worse prognosis of patients with EC (14). Interestingly, MYC interacted physically with DNMT3a, and recruited DNMT3a to gene promoters to induce gene silencing and facilitate DNA methylation (12, 22), thus showing a significant co-expression relationship between the two proteins. Moreover, previous evidence has revealed that DNA methylation greatly affects the expression of tumor suppressor genes or oncogenes (31). Specifically, DNMT3a is identified as a new target of miR-101, and miR-101 overexpression reduces the methylation of the PTEN promoter (24), while DNMT3a may promote PTEN methylation to reduce its expression (16). PTEN is a widely considered as a tumor suppressor that is often inactivated in human cancers, including EC (32). Aberrant expression of PTEN occurs in numerous cancers, and its mutation or deletion is always associated with tumor progression (33). Additionally, PTEN knockdown could promote proliferation but inhibit apoptosis of EC cells (25). Taken together, LINC00470 enhanced the binding relationship between MYC and DNMT3a to reduce PTEN expression by stimulating its methylation, thereby contributing to the malignant behaviors of EC cells such as cell invasion and angiogenesis.

Furthermore, we confirmed that depletion of LINC00470 impeded the oncogenic and angiogenic potentials of EC cells through inhibiting PTEN *in vivo*. Partially consistent with present results, Yan et al., reported that LINC00470 could facilitate the degradation of PTEN mRNA to promote malignant phenotypes of gastric cancer cells (8). Importantly, angiogenesis plays an essential role in tumor development, and its degree is related to tumor grade and aggressiveness (19). As such, we used microvessel density as an important indicator of tumor angiogenesis in our present study. By constructing a nude mouse model of EC, we substantiated that down-regulation of LINC00470 significantly enhanced the PTEN expression but reduced tumor volume and growth rate as well as microvessel density, and that those changes could be restored by down-regulating PTEN, providing *in vivo* evidence for this pathway in EC. Furthermore, in the tumor metastasis model, down-regulation of LINC00470 impeded the metastatic potential of EC cells through increasing PTEN expression.

## Conclusions

In conclusion, this study reports the existence of a novel LINC00470-mediated MYC/DNMT3a/PTEN axis that participates in the occurrence and progression of EC, and presents LINC00470 as a potential therapeutic target for the treatment of EC patients. Given that lncRNAs are increasingly implicated in tumorigenesis, we shall extend the present study to consider the involvement of additional lncRNAs in EC.

## Data Availability Statement

The raw data supporting the conclusions of this article will be made available by the authors, without undue reservation.

## Ethics Statement

We performed all research protocols with the approval of the Clinical Research Ethics Committee of the First Affiliated Hospital of Harbin Medical University and in accordance with the Declaration of Helsinki. All patients had signed informed consent before the experiments. Written informed consent for participation was not required for this study in accordance with the national legislation and the institutional requirements. The animal study was reviewed and approved by First Affiliated Hospital of Harbin Medical University.

## Author Contributions

TY: conceptualization, data curation, formal analysis, supervision, and writing-original draft. YS: conceptualization, data curation, formal analysis, visualization, and writing-original draft. LZ: conceptualization, software, investigation, writing-review, and editing. SW: validation, methodology, writing-review, and editing. JM: resources, investigation, writing-review, and editing. All authors contributed to the article and approved the submitted version.

## Conflict of Interest

The authors declare that the research was conducted in the absence of any commercial or financial relationships that could be construed as a potential conflict of interest.

## Supplementary Material

Supplementary Figure 1 Mechanism map concerning the role of LINC00470 in EC. LINC00470 recruited DNMT3a through MYC to stimulate PTEN methylation and decrease PTEN expression, and overexpression of LINC00470 led to a more aggressive and metastatic cancer phenotype.
