# Supplementary figures and images for: LINC00470 Stimulates Methylation of PTEN to Facilitate the Progression of Endometrial Cancer by Recruiting DNMT3a Through MYC
Source: Front Oncol. 2021 Jun 25;11:646217. doi: 10.3389/fonc.2021.646217 (PMC8267821; doi:10.3389/fonc.2021.646217)

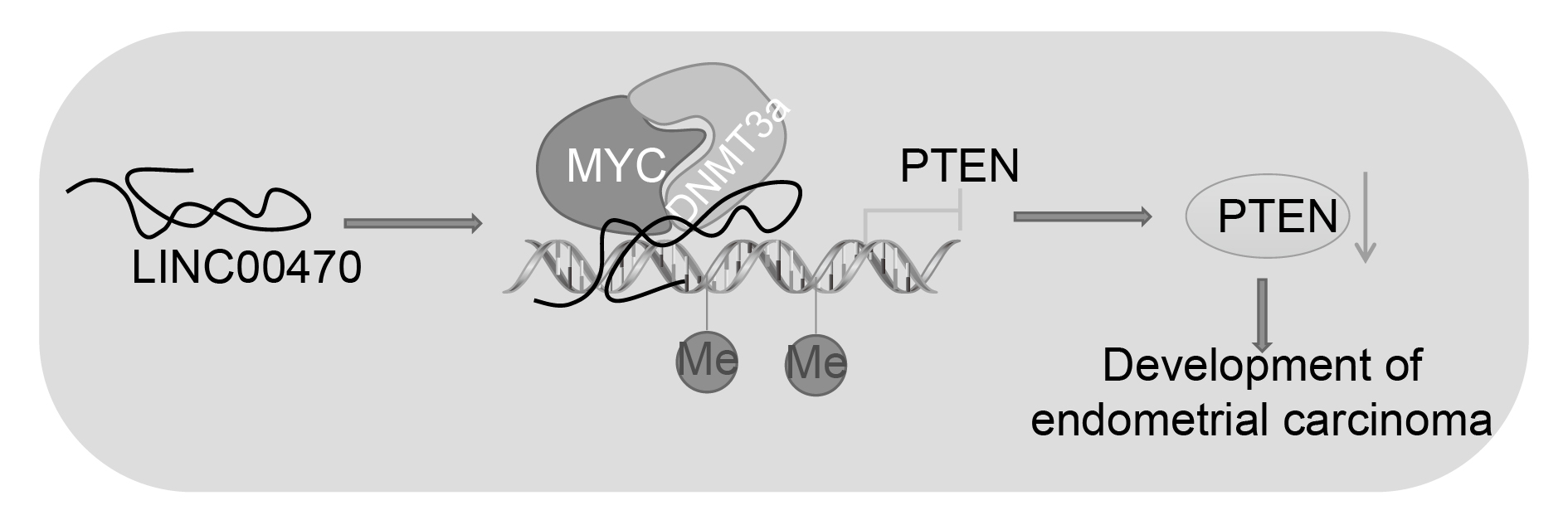

Supplement: Supplementary Figure 1 — Mechanism map concerning the role of LINC00470 in EC. LINC00470 recruited DNMT3a through MYC to stimulate PTEN methylation and decrease PTEN expression, and overexpression of LINC00470 led to a more aggressive and metastatic cancer phenotype. [file Image_1.jpeg]
