# Supplementary material for: LINC00470 Stimulates Methylation of PTEN to Facilitate the Progression of Endometrial Cancer by Recruiting DNMT3a Through MYC
Source: Front Oncol. 2021 Jun 25;11:646217. doi: 10.3389/fonc.2021.646217 (PMC8267821; doi:10.3389/fonc.2021.646217)
Supplement: Supplementary file 2 [file Table_1.docx]

**Table S1** shRNA sequences

| shRNAs | Sequences |
| --- | --- |
| sh-LINC00470#1 | 5′-TCACCGAGCTTATATTTGGTGTGTT-3′ |
| sh-LINC00470#2 | 5′-ACCGAGCTTATATTTGGTGTG-3′ |
| sh-MYC#1 | 5′-GCTTCACCAACAGGAACTATG-3′ |
| sh-MYC#2 | 5′-GGAAACGACGAGAACAGTTGA-3′ |
| sh-MYC#3 | 5′-GGAACTATGACCTCGACTACG-3′ |
| sh-PTEN#1 | 5′-CCACAGCTAGAACTTATCAAA-3′ |
| sh-PTEN#2 | 5′-CTAGAACTTATCAAACCCTTT-3′ |
| sh-PTEN#3 | 5′-CGTGCAGATAATGACAAGGAA-3′ |
| sh-NC | 5′-TTCTCCGAACGTGTCACGT-3′ |

Note: sh/shRNA, short hairpin RNA; NC, negative control.

**Table S2** RT-qPCR primers

|  | Forward primers | Reverse primers |
| --- | --- | --- |
| LINC00470 | 5′-AGACACAGCCTCTACTGTACT-3′ | 5′-CCTCGTCACCTTACGTCAATAC-3′ |
| MYC | 5′-CTTCTCTCCGTCCTCGGATTCT-3′ | 5′-GAAGGTGATCCAGACTCTGACCTT-3′ |
| DNMT3a | 5′-TATTGATGAGCGCACAAGAGAGC-3′ | 5′-GGGTGTTCCAGGGTAACATTGAG-3′ |
| PTEN | 5′-CTGCAGAAAGACTTGAAGGCG-3′ | 5′-GGGAATAGTTACTCCCTTTTTGTC-3′ |
| β-actin | 5′-GGGACCTGACTGACTACCTC-3′ | 5′-AGGGAGGAGCTGGAAGCAG-3′ |

Note: RT-qPCR, quantitative polymerase chain reaction.

**Table S3** ChIP-qPCR primers

|  | Forward primers | Reverse primers |
| --- | --- | --- |
| -2000 to -1700 | 5′-TCCCAGACTGGTGTTGATGC-3′ | 5′-GTTGGATCGCACTCCTACCC-3′ |
| -1700 to -1400 | 5′-CCGAATCAGCTCTCTCACGG-3′ | 5′-CGTGCATTCTCCGATGTTGC-3′ |
| -1400 to -1100 | 5′-CCAGCTCCTTTTCCCACGTT-3′ | 5′-ACACAAGCACCCACATCCAA-3′ |
| -1100 to -800 | 5′-CACGTGACCTCCTTCGGAAA-3′ | 5′-TTTGGGGTTACCGGGTTGAG-3′ |
| -800 to -500 | 5′-ATCCATCCTGCCGGGTTTTC-3′ | 5′-AGAGGATCCCTGTGAGTGGG-3′ |
| -500 to -300 | 5′-AAGGGAGTCGGATGAGGTGA-3′ | 5′-TCGGATCACAATCGTTCGCA-3′ |
| -300 to -0 | 5′-GTATTCCCCTTGCAGGGACC-3′ | 5′-AAAGCTGAGATGGGTGCGTT-3′ |

Note: ChIP, chromatin immunoprecipitation; qPCR, quantitative polymerase chain reaction.
